# Purification, characterization and antifungal activity of LPA a lectin from *Pachira aquatica *seeds: their possible role in plant defence

**DOI:** 10.1186/1753-6561-8-S4-P134

**Published:** 2014-10-01

**Authors:** Ana Luiza Paiva, Ilka Maria Vasconcelos, José Tadeu Abreu Oliveira

**Affiliations:** 1 Federal University of Ceara, Fortaleza, Brazil

## Background

The lectins are an important class of proteins ubiquitous in nature, which plays different roles and functions in biological processes such as recognition molecules within the immune system in animals and as storage proteins or in defence mechanisms against pest and pathogens in plants. Oliveira *et al*. [1] showed that rats fed on a lectin-rich diet from *Pachira aquatica *seeds had a poor growth performance and some internal organs affected. This work reports on the purification and characterization of a lectin from *Pachira aquatica *seeds, with the intention of elucidating its role in plant defence against fungal pathogens.

## Methods

The crude extract of *P. aquatica *seeds was fractionated by precipitation with ammonium sulfate (0-60% saturation) and applied on an Agarose-N-acetyl-D-galactosamine *affinity *chromatography *matrix*. The purified lectin, named LPA, was incubated with three different fungal species to access its effect on spore germination and growth inhibition. Moreover, the resistance of LPA to proteolytic enzymes, a prerequisite of its defense role, was examined.

## Results and conclusion

SDS-PAGE of the matrix-desorbed protein showed a single band with an apparent MW of 65 kDa. The purified lectin (LPA) inhibited the spore germination of *Fusarium solani *and *Fusarium oxysporum *and the vegetative growth of *Colletotrichum gloeosporioides, F. solani *and *F. oxysporum*. LPA also showed to be resistant to proteolysis *invitro*. These results suggest that LPA might contribute to the plant defense against pathogens and constitutes a candidate to improve plant resistance to some phytopathogenic fungi.
